# Microfluidizing Technique Application for Algerian *Cymbopogon citratus* (DC.) Stapf Effects Enhanced Volatile Content, Antimicrobial, and Anti-Mycotoxigenic Properties

**DOI:** 10.3390/molecules28145367

**Published:** 2023-07-12

**Authors:** Amel Boudechicha, Abdelhakim Aouf, Amr Farouk, Hatem S. Ali, Manal F. Elkhadragy, Hany M. Yehia, Ahmed Noah Badr

**Affiliations:** 1 Laboratory of Applied Microbiology, Faculty of Natural and Life Sciences, University of Ferhat Abbas Setif1, Setif 19000, Algeria; amel.boudechicha@univ-setif.dz (A.B.);; 2 Flavour and Aroma Chemistry Department, National Research Centre, Cairo 12622, Egypt; af.mansour@nrc.sci.eg; 3 Food Technology Department, National Research Center, Cairo 12622, Egypt; hatem.owyean1@gmail.com; 4 Biology Department, College of Science, Princess Nourah bint Abdulrahman University, P.O. Box 84428, Riyadh 11671, Saudi Arabia; 5 Food Science and Nutrition Department, College of Food and Agriculture Science, King Saud University, P.O. Box 2460, Riyadh 11451, Saudi Arabia; 6 Food Science and Nutrition Department, Faculty of Home Economics, Helwan University, Helwan 11611, Egypt; 7 Food Toxicology and Contaminants Department, National Research Centre, Dokki, Cairo 12622, Egypt

**Keywords:** aflatoxins, antibacterial, anti-mycotic, cytotoxicity, microfluidization, mycotoxin reduction, volatile constituent changes, ochratoxin

## Abstract

Medicinal plant extracts are a promising source of bioactive minor contents. The present study aimed to evaluate the distinguished volatile content of Algerian *Cymbopogon citratus* (DC.) Stapf before and after the microfluidization process and their related antimicrobial and anti-mycotoxigenic impacts and changes. The GC-MS apparatus was utilized for a comparative examination of Algerian lemongrass essential oil (LGEO) with its microfluidization nanoemulsion (MF-LGEO) volatile content. The MF-LGEO was characterized using Zetasizer and an electron microscope. Cytotoxicity, antibacterial, and antifungal activities were determined for the LGEO and MF-LGEO. The result reflected changes in the content of volatiles for the MF-LGEO. The microfluidizing process enhanced the presence of compounds known for their exceptional antifungal and antibacterial properties in MF-LGEO, namely, neral, geranial, and carvacrol. However, certain terpenes, such as camphor and citronellal, were absent, while decanal, not found in the raw LGEO, was detected. The droplet diameter was 20.76 ± 0.36 nm, and the polydispersity index (PDI) was 0.179 ± 0.03. In cytotoxicity studies, LGEO showed higher activity against the HepG2 cell line than MF-LGEO. Antibacterial LGEO activity against Gram-positive bacteria recorded an inhibitory zone from 41.82 ± 2.84 mm to 58.74 ± 2.64 mm, while the zone ranged from 12.71 ± 1.38 mm to 16.54 ± 1.42 mm for Gram-negative bacteria. Antibacterial activity was enhanced to be up to 71.43 ± 2.54 nm and 31.54 ± 1.01 nm for MF-LGEO impact against Gram-positive and Gram-negative pathogens. The antifungal effect was considerable, particularly against *Fusarium* fungi. It reached 17.56 ± 1.01 mm and 13.04 ± 1.37 mm for LGEO and MF-LGEO application of a well-diffusion assay, respectively. The MF-LGEO was more promising in reducing mycotoxin production in simulated fungal growth media due to the changes linked to essential compounds content. The reduction ratio was 54.3% and 74.57% for total aflatoxins (AFs) and ochratoxin A (OCA) contents, respectively. These results reflect the microfluidizing improvement impact regarding the LGEO antibacterial, antifungal and anti-mycotoxigenic properties.

## 1. Introduction

Essential oils are a mixture of naturally highly volatile and aromatic hydrophobic components produced from various plant parts [1]. *C. citratus* (lemongrass) is a source of essential volatile constituents and is utilized frequently in foods, cosmetics, and traditional medicine. New types of *C. citratus* may be characterized by distinguished activities linked to their unique contents. The essential oil causes lemongrass’s biological action due to citral, the predominant content of lemongrass essential oil (LGEO), which is responsible for the oil’s potent biological activities [2,3]. The LGEO, also known as lemonal, belongs to monoterpenoids, with diastereo-isomer of E-isomer recognized as geranial or citral A and a Z-isomer is recognized as neral or citral B. Due to cis–trans isomerism at the C=C link at the aldehyde group, the commercial citral is formed as a mixture of two isomers. The geranial has a distinct aroma reminiscent of lemon, while the neral has a less prominent scent. These isomers have potent antimicrobial properties [4] and can be applied in insecticidal, pheromonal, pharmaceutical, and cosmeceutical applications [5]. Again, citral is a fundamental intermediate required for the production of various flavoring and aroma components, including ionones, methyl ionones, and vitamins A and E [6,7]. The LGEO showed a remarkable antifungal efficacy against pathogenic strains of *Aspergillus niger* and *Fusarium oxysporum*, while it has a low effect on *Aspergillus flavus* [8]. The *Fusarium* genus is notorious for damaging plants, causing financial and agricultural losses, and producing mycotoxins in cereal crops. At a concentration of 250 ppm, LGEO wholly inhibited spore germination; the IC_50_ value was determined to be 0.98 ppm.

Natural resources, such as essential oils, were utilized to control fungal growth and limit aflatoxin production. These actions may be due to their potential antifungal and anti-carcinogenic properties, aflatoxin (AFs) generation and reduction, and consumer demand for quality and safety in food additives [9]. However, their low water solubility and strong aroma make them difficult to incorporate into pharmaceutical preparations and food products, even at high microbiologically effective doses [10]. Mycotoxins, the secondary metabolites that fungi create and can contaminate food supplies and animal feeds, result in disorders called mycotoxicosis that can be fatal. Aflatoxin B_1_ (AFB_1_) is the most potent hepatocarcinogen known among the four primary forms of aflatoxins (AFB_1_, AFB_2_, AFG_1_, and AFG_2_). Acute aflatoxicosis can result in nausea, vomiting, edema, and even death [11].

Moreover, other mycotoxins, such as ochratoxins, may cause several illnesses and public issues due to their presence in human food or animal feed [11]. These mycotoxins cause serious health issues, from acute poisoning to chronic diseases, including tissue damage, cancer, and immune system disorders [12]. Mycotoxins research is a complex investigation; however, it possesses significant hazards related to food safety and human health. The mycotoxins challenge is joined to their difficulty of detection (minimal quantities) and because they may become masked by food compounds [13]. The novel strategies to solve these issues include phytochemical applications to reduce mycotoxin contamination [14]. These natural components may be applied as extracts or after technological applications such as nanoemulsions [15,16].

As a result, the nanoencapsulation of essential oils may be a viable alternative for their wide applications. Essential oil nanoemulsions between 60 and 600 nm droplet sizes have enhanced food components’ stability, flavor, appearance, nutritional value, and biological activity [17]. However, there are high-energy and low-energy nanoemulsion manufacturing methods. The second category is typically more likely to be used in the food industry due to their scale-up, equipment accessibility, and capacity to create nanoemulsified systems without organic solvent utilization [18]. Microfluidization, a high-energy emulsification technique, has produced effective nanoemulsion-based strategies in several research projects, with average sizes below 200 nm, like in clove oil (30.76 nm), fennel oil (8 nm) and fish oil (155 nm) [1,19,20]. According to the definition of micro fluidization processing, a coarse emulsion is pumped under high pressure via an interaction chamber with two flow channels [21].

For LGEO, the impact of processing conditions on oil–alginate nanoemulsions’ physicochemical properties was studied [22]. In addition, microfluidization was used to enhance the antimicrobial activity of the oil against *Escherichia coli* and *Botrytis cinerea* [23,24]. No studies have been conducted on controlling fungal growth, decreasing aflatoxin formation, or cytotoxicity utilizing emulsions developed with LGEO manufactured using a microfluidizer. Furthermore, no information was provided on converting LGEO into nanoemulsion forms, cytotoxicity, or antifungal effects. Therefore, this study aimed to evaluate the impact of the microfluidization process on the lemongrass essential oil (LGEO) nanoemulsion cytotoxicity, besides the assessment of the enhancement of the antibacterial, antifungal, and anti-mycotoxigenic properties. The bioactivity of microfluidized nanocapsules can be altered by penetration properties and LGEO activity. This effect was determined by investigating the physical characteristics of the formed nanoemulsions and the influence of micro fluidization on the volatile elements of the LGEO. Also, the applied experiment was conducted using a simulated medium containing a spore suspension of toxigenic fungi.

## 2. Results

### 2.1. Effect of Microfluidization on LGEO Volatiles

The dried aerial parts submitted to hydrodistillation produced a light-yellow oil yielding 1.76 ± 0.05%. The identification of chemical composition using GC-MS revealed the presence of 27 constituents, representing 97.73% of the total hydrodistilled LGEO content (Table 1).

**Figure 1:**
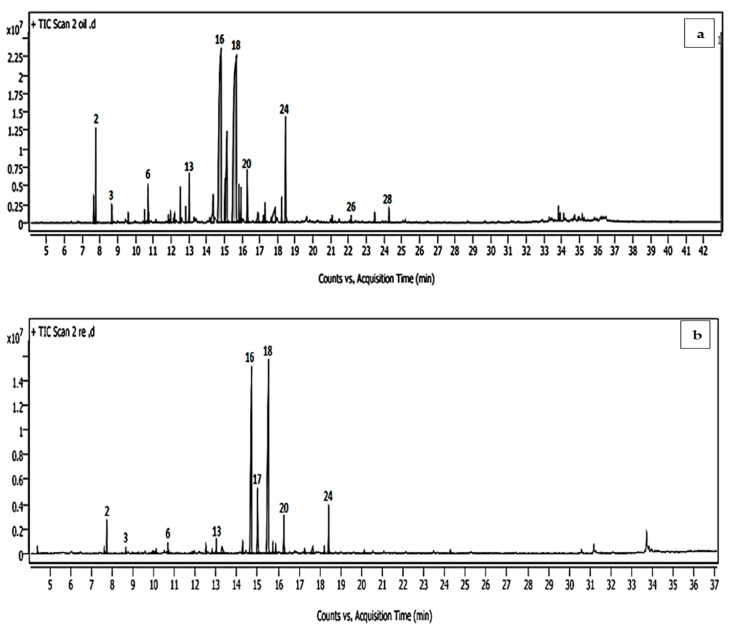
Volatile chromatograms for (**a**) LGEO hydrodistilled oil and (**b**) microfluidized LGEO nanoemulsion. LGEO: lemongrass emulsion of essential oil; MF-LGEO: microfluidizing emulsion of lemongrass essential oil.

Neral and geranial were the predominant compounds, with 26.91 and 30.73%, respectively, followed by geraniol (9.69%), geranyl acetate (5.06%), and *β*-myrcene (3.61%). The nanoemulsion chemical analysis of the LGEO produced by the microfluidization technique showed a quantitative difference compared to the content of the raw LGEO volatile oil (Table 1). However, some compounds of minor content were found to be absent from the LGEO volatile content by nanoformation technique (camphor, citronellal, bergamotene, α-farnesene, and caryophyllene oxide) and also in new components detected in MF-LGEO (decanal) (Table 1; Figure 1). Identified components in the nanoemulsion were represented in 97.53% of the total nanoemulsion oil. Similar to the hydrodistilled LGEO, geranial (35.48%) and neral (28.95%) were predominant, followed by geraniol and geranyl acetate (7.97 and 4.55%, respectively). Several mono- and sesquiterpenes, such as camphor, citronellal, bergamotene, α-farnesene, and caryophyllene oxide, in the nanoemulsion LGEO when compared to the hydrodistilled oil.

### 2.2. Droplet Size, PDI, and ξ-Potential

**Figure 2:**
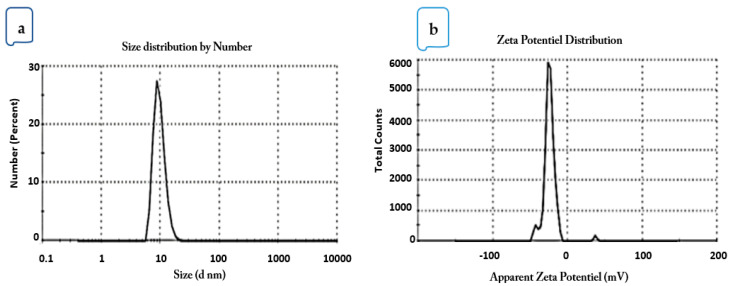
(**a**) Particle size distribution and (**b**) ξ-potential of MF-LGEO nanoemulsion.

The particle size and PDI of the microfluidized LGEO nanoemulsions were determined. The results showed that the mean droplet diameter of the nanoemulsions was 20.76 ± 0.36 nm and PDI of 0.179 ± 0.03 (Figure 2a). The interfacial electrical charge of the droplets after microfluidization processing was −23.9 mV (Figure 2b).

### 2.3. Transmission Electron Microscopy (TEM) Images

Transmission electron microscopy (TEM) is a technique that is frequently used to validate droplet-size findings. The emulsion droplets had the appropriate nanometric diameter and were spherical. Likewise, the results of the homogenized emulsion’s transmission electron micrograph nearly matched those of the results for droplet size. The results from the dynamic light scattering (DLS) and TEM varied slightly because TEM produced a slightly lower value, which is expected as shrinkage may occur when samples are dried for TEM investigation (Supplementary Figure S1).

### 2.4. Cytotoxicity of LGEO and Its Microfluidized Nanoemulsion

The presumed cytotoxic effects of LGEO and its microfluidized nanoemulsions against HepG2, Vero, and WI-38 were investigated in this study using MTT and WST-1 viability assays. Cisplatin was used as a reference drug. Table 2 shows a reduction in the cell viability percentage of HepG2 after treatment with LGEO and its nanoemulsions, compared to the treated Vero and WI-38 cell lines. The oil exhibited the highest growth inhibitory activity against the HepG2 cell line with the lowest IC_50_ (1.78 and 28.54 μg/mL) compared to cisplatin (IC_50_ 20.71 and 40.95 μg/mL) for both assays used, MTT and WST-1, respectively. The lower IC_50_ values of HepG2 cells compared to the Vero and WI-38 cell lines show the selectivity of the studied LGEO (Table 2).

**Figure 3:**
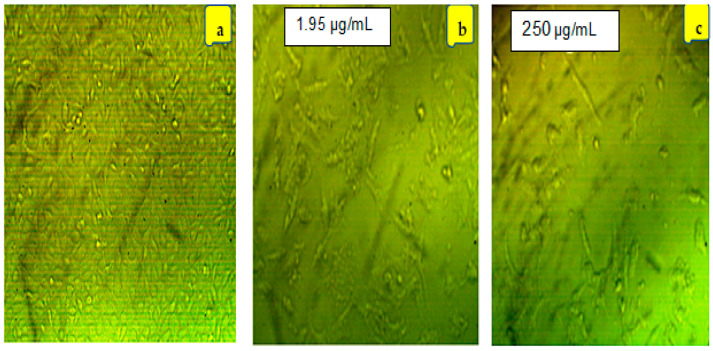
Cellular morphology changes of the HepG2 cells exposed to (**b**) LGEO and (**c**) microfluidized LGEO nanoemulsion compared to (**a**) The control using 40× Zeiss Axio Vert A1 microscope.

It was discovered that the morphological changes in HepG2 cell lines were dependent on concentration. Figure 3 depicts the cell-morphological changes at concentrations close to the IC50 for both LGEO and nanoemulsion. Compared to controls, cells exposed to various doses of LGEO and its nanoemulsion after 24 h showed a decrease in normal morphology and cell adhesion capacity (Figure 3). HepG2 cells underwent morphological changes as they were shrunken and appeared smaller. Also, it was observed that compared to the control group, gaps between cells were enlarged, and cells had an irregular appearance with a rounded form. Moreover, some cells shrunk at concentrations below the IC_50_ and developed a torn membrane, indicating apoptotic cell death. As the amounts of LGEO and nanoemulsion rose, the percentage of aberrant and dead cells also rose. The impact of different concentrations for the LGEO and MF-LGEO on the cell line viability was provided in Supplementary Figure S2.

### 2.5. Antimicrobial Effect of LGEO and MF-LGEO

#### 2.5.1. Antibacterial Effect of LGEO and MF-LGEO

**Figure 4:**
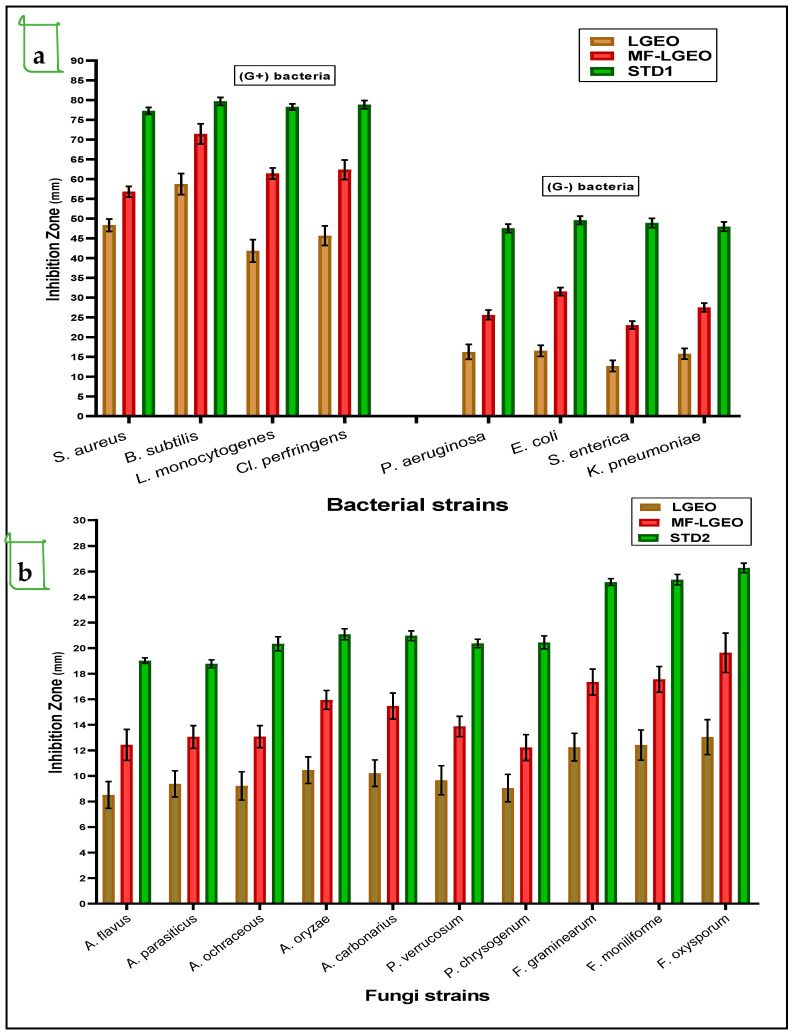
Antibacterial and antifungal activities of lemongrass oil and its nanoemulsion using well-diffusion assay. The data are expressed as means ± SD (where n = 3, *p* ≤ 0.05); SD: standard deviation. LGEO: lemongrass essential oil; MF-LGEO: microfluidizing nanoemulsion of lemongrass essential oil; STD1: ciprofloxacin (standard antibiotic) applied as a positive control for bacteria; STD2: Nystatin used as control positive antifungal compound. Figure 4a represents the antibacterial effect of LGEO and MF-LGEO against Gram-positive and Gram-negative strains. Figure 4b represents the antibacterial effect of LGEO and MF-LGEO against toxigenic fungal strains.

Regarding the activities differentiation between LGEO and MF-LGEO, they were evaluated for their antimicrobial impacts using the well-diffusion assay. Firstly, the antibacterial activities against Gram-positive strains of pathogenic bacteria were more efficient than those recorded against Gram-negative ones (Figure 4a), even for the standard antibiotic. The antibacterial activity of the MF-LGEO was recorded more than LGEO activity. Moreover, in some cases, it was close to the effect of the standard antibiotic (such as against *B. subtilis*). It was noticed that Gram-negative strains of bacteria showed more resistance to the antibacterial effect of both treatments and traditional antibiotics. This resistance may be linked to the nature of the cell wall structure of the Gram-negative strains. The strain of *S. enterica* was recorded as the lowest-influenced strain by the treatments of LGEO and MF-LGEO. Also, the variation in the influence of the LGEO and MF-LGEO as an antibacterial was most straightforward for the Gram-positive strains of the applied pathogens. Its high content of bioactive compounds, such as citral, geraniol, and limonene, is predominantly responsible for the antibacterial effect of LGEO; it is an effective natural treatment for bacterial infections due to the potent antimicrobial properties possessed by these compounds. The essential oil of *C. citratus* is effective against various bacteria, including Gram-positive and Gram-negative strains. The development of several bacteria, including *Escherichia coli* (*E. coli*), *Staphylococcus aureus* (*S. aureus*), *Salmonella enterica* (*S. enterica*), and *Pseudomonas aeruginosa* (*P. aeruginosa*), is efficiently inhibited by it in invitro studies. 

#### 2.5.2. Antifungal Effect of LGEO and MF-LGEO

Concerning the antifungal effect of LGEO and MF-LGEO, the results reflect an efficiency against the applied strains of toxigenic fungi (Figure 4b). More antifungal efficiency was shown against the *Fusarium* strains. It is worth mentioning that MF-LGEO showed more inhibition impact against toxigenic fungal strains than LGEO. This impact may link to the encapsulation process’s preservation effect for the oil’s bioactive component that protects its breakdown. Also, the efficiency of the LGEO and MF-LGEO to inhibit the growth rate of five *strains* of the *Aspergillus* genus was shown against *A. flavus, A. parasiticus, A. ochraceus, A. oryzae,* and *A. carbonarius.* The antifungal effect of MF-LGEO was recorded close to the standard antifungal of Nystatin and was more than the LGEO antifungal effect. 

#### 2.5.3. Anti-Mycotic and Anti-Mycotoxigenic Effect

The anti-mycotic effect of the LGEO and MF-LGEO is shown in Table 3, where its ability to reduce the mycelia growth of two strains in liquid media is demonstrated. The results reflected a more significant inhibition effect for MF-LGEO than LGEO. For the strain of *A. flavus*, the spores could grow by 59.74% and 26.54% of the controlled growth by the presence of the LGEO and MF-LGEO, respectively, in the fungal-growth media. This inhibition effect was also clear for the media containing *A. carbonarius* spores. The growth rate of the spores for media including the LGEO and MF-LGEO was recorded as 56.04% and 23.59%, respectively, compared to the complete growth in the control treatment. The reduction in the growth rate using MF-LGEO as most ratios could be linked to improving terpene content, including neral and geranial contents.

The minimal fungicidal concentration (MFC) values were more enhanced for MF-LGEO than LGEO against the fungal spores of *A. flavus*. This ameliorative effect may be linked to the encapsulation process provided by nanoemulsion formation. The same enhancement was recorded for applying the LGEO and MF-LGEO against the fungal spores of *A. carbonarius.* Hence, *A. flavus* fungi can produce aflatoxins, the total content of AFs determined in the liquid growth media. A reduction of AF production was more clearly shown in MF-LGEO than LGEO—the AF content order is Nystatin (standard antifungal) < MF-LGEO < LGEO < the control. The AF reduction ratios are 54.3% and 44.82% for MF-LGEO and LGEO, respectively. This reduction ratio reaches 74.57% and 46.53% for the MF-LGEO and LGEO, respectively, for the OCA production in the media containing *A. carbonarius* spores.

Anti-mycotic and anti-mycotoxigenic activities have been reported for the LGEO, suggesting it may be helpful as a natural treatment for fungal infections and diseases caused by mycotoxins. According to scientific studies, essential oil from the LGEO has shown promise as an anti-mycotic agent. The LGEO can provide alternative solutions for fungal infections, mainly those related to mycotoxigenic fungi. Algerian lemongrass is investigated for its potential to inhibit aflatoxin production. Anti-aflatoxigenic activity, effectively reducing aflatoxin production in fungal cultures, were the parameters that were evaluated in liquid media. The mechanism of action is not yet fully understood, but the Algerian lemongrass compounds are believed to interfere with fungal metabolism, ultimately suppressing aflatoxin synthesis. It is worth noting that while the present type of Algerian lemongrass demonstrates promising anti-mycotic and anti-aflatoxigenic effects in laboratory studies, further research is needed to validate its efficacy in clinical settings and determine the optimal dosage and delivery methods.

## 3. Discussion

Modern research pointed out that natural components obtained from plants are authorized to be applied in food applications to benefit from their supportive antifungal effects [25]. Numerous of these components have been found to possess hopeful antifungal impacts against *Aspergillus* fungi, including neral, citral, geraniol, thymol, geranial, and cinnamaldehyde [26,27]. Thus, their application for food preservation will achieve many promising impacts. Moreover, using modern technology, such as microfluidization, to form bioactive nanoemulsions may enhance this activity and lead to intelligent application forms with economic outcomes [28,29]. Using this technological application, the activity of the bioactive content can be improved, besides the presence of new bioactive derivatives. Another point is that the new bioactive molecules’ size will increase their activity and penetration ability into the fungal cell wall and alter its structure. This previous effect will improve the antifungal efficiency of such bioactive components.

The quality and quantity of the extracted oil depend on many factors, including cultivation area and its environmental conditions, stage of maturity, and extraction techniques [3]. Based on the same extraction technique, the obtained yield of the current study is higher than those extracted by Boukhatem et al. [30] and Benoudjit et al. [31] from the Blida region in the north of Algeria with 0.6 and 0.8% (*v*/*w*), respectively. To our knowledge, nothing was reported in the literature concerning the LGEO of Bousaada, M’Sila Province, Algeria. From the qualitative point of view, the above findings are in agreement with previous studies [31] that reported neral and geranial as the significant components of LGEO but with quantitative differences. Meanwhile, geraniol was detected in the current study with a substantial predominance compared to *β*-myrcene, which followed geranial and neral concentrations in Blida LGEO [30,31].

High-pressure homogenization is an energy-intensive approach that decomposes some of the active components of essential oils while accumulating others [32]. For example, the predominance of thymol and carvacrol in the Algerian *Saccocalyx satureioides* oil nanoemulsion was at the expense of borneol and α-terpineol concentrations detected in the hydrodistilled oil [33]. Meanwhile, an inverse relationship was reported by Ali et al. [32], while borneol and α-terpineol were reported as predominating in the nanoemulsion during the encapsulation of the *Origanum glandulosum* oil. However, based on the present study’s findings, the same qualitative volatiles profile for the LGEO could be observed after formulating nanoemulsion using the microfluidization technique with quantitative differences but without changes in the predominates. Moreover, the oil and citral content in the microfluidized nanoemulsion was recorded by increment. These compounds were previously identified by high antifungal activity [34]. In this regard, this technological process can recommend improving the oil application for microbial safety of such food products.

With an increase in processing pressure and cycle count, the average droplet size of nanoemulsions decreased [22]. These outcomes concur with those previously reported in nanoemulsions based on curcumin or pea protein [35,36]. The smallest average droplet size was usually seen in nanoemulsions processed at 150 MPa [10]. Nothing was reported in the literature concerning the effect of microfluidization on the volatile constituents of essential oils. Following the results of our study, Pilong et al. [19] reported a higher content of eugenol (70.69%) after microfluidized clove essential oil in nanoemulsion form was compared to the untreated essential oil (60.11%), which might be attributed to the high surface area/volume ratio of eugenol, which occurred through the plenty amount of fine clove essential oil droplets. Meanwhile, other constituents in clove oil decreased upon microfluidization, like benzyl alcohol and caryophyllene. They advised the necessity of a specific mechanistic study in this area. Several studies have posited that the emulsion’s physical stability and biological activity may be altered by Ostwald ripening, flocculation, or coalescence [37].

Compared to the droplet sizes of eugenol and clove nanoemulsions, the size of LGEO by microfluidization was recorded as more minor. This can be attributed to the higher polarity of eugenol, which enhances its solubility in the aqueous phase compared to the isomers of citral [24]. The molecular structure, volatile compound content, interfacial tension, or surfactant affinity of different essential oils or their primary compound-loaded nanoemulsions may all be responsible for variations in droplet size [17]. The average droplet size of corn oil nanoemulsions processed once by microfluidization at various pressures was drastically reduced, down to 165 nm, according to Qian and McClements [38]. At the same time, further cycles through the microfluidizer had little impact. A few studies have recently been published on creating essential oil-infused nanoemulsions using microfluidization. According to Donsì et al. [39], nanoemulsions containing D-limonene with a combination of terpenes treated for ten cycles at 300 MPa had droplet diameters ranging from 74.4 to 356.7 nm. However, the collision and coalescence of droplets during nanoemulsion production due to Brownian solid movement and sluggish surfactant adsorption may cause the droplet size to increase with increased operating pressure [21].

The PDI values below 0.2 indicate uniformity among oil droplet sizes or monomodal distributions, as shown in Figure 1a, and therefore better stability [15]. The previous findings agreed with Salvia-Trujillo et al. [10,22], who microfluidized LGEO with sodium alginate and Tween 80 with a droplet size between 7.1 and 7.35 nm, while the PDI was 0.34. In contrast, Gago et al. [24] recorded a higher size (30.25–36.45 nm) and more PDI (0.65–0.78) for the LGEO droplets microfluidized using sodium alginate and Tween 80, compared to the results of the current study. The mean droplet diameter and PDI of the nanoemulsions were found to be affected by the operating pressure. Due to the strong created disruptive force, it has often been reported that employing increased operating pressure causes emulsion droplet sizes to decrease. Tween 80 can reduce the friction at the oil–water interface, which lowers the free energy required to generate nanoemulsions and encourages the creation of tiny droplets with a low PDI and narrow size distribution. The major LGEO components are neral and geranial, which represent the cis and trans-isomers of citral. Each isomer’s topological polar surface area is 17.07 Å2, indicating a lower polarity and smaller size than polar volatiles like eugenol (164.08 g/mol and 29.46 Å2, respectively) [40].

According to Salvia-Trujillo et al. [22], passing the coarse emulsion through the microfluidization system significantly reduced the droplet ξ-potential regardless of the pressure applied, which meant an increase in the net electrical charge of LGEO droplets. Generally, particles with ξ-potential more negative or positive than ±30 mV are usually considered stable since the electrical charge of the droplets is strong enough to assume that the repulsive forces between droplets are predominant in the nanoemulsion [17]. Moreover, CMC, an anionic hydrocolloid, gives these nanoemulsions significant negative ξ-potential. Since they can be absorbed into the interfacial layer, the anionic groups in the polymer chain of CMC and their surface characteristics can stabilize the nanoemulsions. However, their stabilizing activity depends on potential interactions and competition between previously adsorbed species [36]. A nonionic emulsifier/surfactant, such as Tween 80, can impart a negative charge to oil droplets due to the preferential adsorption of hydroxyl ions from the aqueous phase or the presence of anionic impurities like free fatty acids in the surfactant or oil phases [18]. Differences observed in the ξ-potential of emulsions and nanoemulsions formulated with different essential oils might be attributed to differences in the dissociation degree and the number of ionizable oils compounds [41]. Therefore, LGEO nanoemulsion exhibited lower negative ξ-potential values than clove oil or pure eugenol [24]. This mechanism can illustrate the improvement recorded for the MF-LGEO and may support its more expected activity by application.

The cytotoxic impacts recorded for the present MF-LGEO agreed with Trang et al. [42], where positive cytotoxic activities were shown in *C. citratus* oils collected from different locations in Vietnam against lung cancer cells, with an IC_50_ range of 4.25–8.93 μg/mL. Citral (neral and geranial), the primary component of LGEO, was reported to have cytotoxic activity against multiple human leukemia cell lines and to cause apoptosis in leukemia cells by activating procaspase-3 [43]. Geraniol, the second abundant substance, has been shown to have considerable anticancer action through various signaling pathways [44]. When the cytotoxic capacities of LGEO and its nanoemulsion are compared, it is seen that nanoemulsions showed lower capacity, which is attributed to the small amount of LGEO in the formulation of nanoemulsions. This is in agreement with Verma and Preet [45], where the maximum percent cell survivability of HEK293 cell lines reached 61% at the highest after exposure to 100 ppm of LGEO nanoemulsion for 48 h. The changes recorded for the volatile contents after the microfluidizing process may be linked to the safety characteristics reflected in the cytotoxicity results. As neral and geranial are quantitatively increased, anti-inflammatory properties have previously been reported [27].

The LGEO’s ability to suppress biofilm formation may make it useful in various settings, including wound care and dental health, by reducing bacterial colonization and persistence on surfaces [46]. In contrast, other research pointed out the LGEO functionality to prevent the production of bacterial biofilms. Bacterial colonies, known as biofilms, form on surfaces and are notoriously difficult to remove due to antibiotic resistance [47]. Moreover, the LGEO contains bioactive substances that may break the bacterial cell membrane, resulting in intracellular content leakage and cell death. Its bactericidal efficacy against a wide range of bacteria is assumed to come from this mechanism of action [48]. The LGEO has been shown to have anti-inflammatory properties, which may help explain why it is effective against germs [49]. Reduced inflammation, a typical reaction to bacterial infection, may explain why the LGEO relieves symptoms and speeds recovery [50]. The novel trend of the LGEO application was linked to its synergistic effect with other antimicrobial agents. Research reported that the antibacterial effects of other antimicrobial agents might be boosted by combining them with the LGEO [51]. This data supports the idea that using the LGEO in conjunction with conventional antibiotics may improve the efficacy of both treatments while decreasing the likelihood of antibiotic resistance. The citral, geraniol, and limonene components are just a few of the bioactive chemicals found in LGEO that have been shown to have potent antifungal action against various kinds of fungi [52,53]. The most prevalent type of *Candida* to cause infections in humans, *Candida albicans*, has been found in previous research to be efficiently inhibited by the LGEO [54,55]. The LGEO has been shown to have antifungal action against various fungi, including the potentially fatal *Cryptococcus neoformans* and the common athlete’s foot fungus, *Trichophyton mentagrophytes* [52].

Several fungal species, including *Candida*, dermatophytes, and *Aspergillus*, are inhibited by the LGEO. Human infections caused by these fungi often resist treatment with standard antifungal medications. Bioactive chemicals, including citral, geraniol, and limonene, are thought to be responsible for the LGEO’s anti-mycotic and anti-mycotoxigenic properties [52]. These substances have been proven to interfere with the metabolic activities of fungi, slow their development, and destroy their cell membranes. The capacity of the LGEO to fight against fungal infections and mycotoxin synthesis may be due, in part, to the antioxidant and anti-inflammatory properties it has. Several studies have investigated the antifungal properties of lemongrass and its essential oil [30,31]. Lemongrass oil has shown inhibitory effects against various fungi, including *Candida albicans* and *Aspergillus niger*. These fungi are responsible for causing common infections like candidiasis and dermatophytosis. The anti-mycotic properties of lemongrass are attributed to its active constituents, such as citral, geraniol, and myrcene, which exhibit fungicidal or fungistatic effects.

LGEO’s antifungal properties are likely due to a combination of factors. Fungi may die due to membrane disruption, allowing intracellular contents to flow [56]. It may also disrupt the metabolic activities of fungal cells, preventing them from multiplying and spreading [57]. In addition to its antifungal action, the antioxidant and anti-inflammatory properties of the LGEO essential oil are recorded in the cytotoxicity evaluation. They can play a role in mitigating oxidative stress and inflammation caused by fungal infections. Moreover, the application of *L. petersonii* essential oil against microbial fungi reflects a high activity, which was explained by the joint neral and geranial content.

Improvement recorded in the LGEO activity by the microfluidization process can be linked to the advancement of such neral, geranial, and decanal components. These components were increased in the content of the MF-LGEO nanoemulsion. The study of Kim et al. [58] pointed out the neral and geranial impact of *L. petersonii* essential oil as fumigants inhibited the fungal strain of *Aspergillus*. These compounds were classified as significant antifungal compounds. Also, the functionality of decanal as an active antifungal compound was reported before [28,59]. Decanal and its derivatives are considered natural weapons of plants against harmful fungi infection [59]. The present study referred to the presence of decanal in the MF-LGEO due to the microfluidization process, besides the recording of neral, geranial, dihydro-linalool acetate, and carvacrol increments in quantities (Table 1), where all these compounds are possessing a unique antifungal activity that can explain the inhibition of fungi growth recorded in Figure 3b, as well as the mycotoxin reduction shown in Table 3.

## 4. Materials and Methods

### 4.1. Chemicals and Microorganisms

All of the chemicals applied in this investigation were of HPLC- grade and were purchased from Sigma-Aldrich, Saint Louis, MO, USA. The HepG2 cell line, Vero normal cell line (originated from the African green monkey kidney), and WI-38 normal lung cells were purchased from the American Type Culture Collection (ATCC)^®^ through VACSERA (Cairo, Egypt). The dimethyl sulphoxide (DMSO) was obtained from Merck, Darmstadt, Germany. The fetal calf serum (FCS) and penicillin, Ciprofloxacin, and streptomycin were obtained from Hyclone, Logan, UT, USA, and Dulbecco’s Modified Eagle Medium (DMEM) was purchased from Gibco, Thermo Fisher Scientific, Inc., Waltham, MA, USA.

The antibacterial assay was made using LGEO and MF-LGEO against bacteria of Gram-positive and Gram-negative strains provided by the Laboratory of Applied Microbiology, Ferhat Abbas University, Setif, Algeria. The Gram-positive strains were *Staphylococcus aureus* ATCC 6538P, *Bacillus cereus* ATCC 14579, *Listeria Monocytogenes* ATCC 7644, and *Clostridium perfringens* ATCC 13124. The Gram-negative strains used for the antibacterial evaluation were *Pseudomonas aeruginosa* ATCC 27853, *Klebsiella pneumoniae* ATCC 13883, *Salmonella enterica* ATCC 13076, and *Esherishia coli* ATCC 7839.

The antifungal assay was determined against the fungi strains of *A. flavus* ITEM 698, *A. parasiticus* ITEM 11, *A. carbonarius* ITEM 5010, *A. ochraceus* ITEM 5117, *A. oryzae* ITEM B5, *Penicillium verrucosum* NRRL 695, *P. chrysogenum* ATCC 48271, *Fusarium graminearum* ATCC 56091, *F. moniliforme* ITEM 52539, and *F. oxysporum* ITEM 12591. The ITEM provided these strains—agro-food microbial culture collection, ISPA, CNR, Bari, Italy.

### 4.2. Extraction of LGEO Essential Oil by Hydrodistillation

The Algerian lemongrass (*Cymbopogon citratus* (DC.) Stapf) aerial parts were harvested from Bousaada, M’Sila Province, between the Saharan Atlas Mountains and the el-Hodna Salt Lake (north-central Algeria). A taxonomist at the Department of Biology and Plant Ecology (Faculty of Life Sciences and Nature, Ferhat Abbas University, Setif 1, Al-geria) identified the plant materials and deposited them in the herbarium with a voucher specimen bearing the Algerian number CAS28/06/21. They were then allowed to dry at room temperature in a dark and dry place. A Clevenger-style device was used to hydrodistill the dried aerial portions of *C. citratus* (DC.) Stapf for three hours to obtain the essential oil. The essential oils were extracted, dried with anhydrous sodium sulfate, and kept (at −20 °C) until analysis in airtight glass vials sealed with aluminum foil [60]. The extraction experiment was repeated three times.

### 4.3. Preparation of LGEO nanoemulsion

Food-grade carboxymethyl cellulose (CMC) emulsion was prepared in warm water (2% *w*/*v*; 2 h) using a mechanical stirrer until complete solubility. The coarse emulsion was prepared according to Salvia-Trujillo et al. [22]; it was briefly made by mixing the CMC solution (2%) as the aqueous phase and essential oil (1% *v*/*v*) as the lipidic phase plus Tween 80 (1% *v*/*v*) as a surfactant, with a magnetic stirrer for 30min. The final solution was fed to a microfluidization system to obtain the nanoemulsion form of the prepared coarse solution of the essential oil encapsulated by the CMC. The coarse emulsion was passed through the system five times to ensure homogeneity and stability for the final solution. The applied condition of the microfluidizer (M110P, Microfluidics, Westwood, MA, USA) was 150 MPa, five cycles. The coarse emulsion was refrigerated using an external cooling coil immersed in ice at the outlet point of the interaction chamber to keep the product temperature at no more than 15 °C. The final emulsion after the applied cycles was kept cooling until further investigations.

### 4.4. Nanoemulsion Characterizations

Zeta Sizer Nano ZS (Nano-S90, Zetasizer, Malvern Panalytical Ltd., Enigma Business Park, Grovewood Road, Malvern, UK) was used to assess particle size distribution, ξ-potential, and polydispersity index (PDI) at 25 ± 0.1 °C [61].

The emulsion was put into a 50 mL graduated bottle, sealed, and kept at 25 °C for five days. The emulsion stability index was determined through the emulsion serum separation. The formula %ES = (H1/H0) × 100 was used, where ES is the emulsion stability, H1 is the upper phase height, and H0 is the starting emulsion height [9].

The morphology of nanoemulsions and nanocapsules was studied using transmission electron microscopy (JED 1230, JEOL Ltd., and Tokyo, Japan). The samples were examined using a 160 kV operation [32]. Twenty microliters of the diluted sample were applied to a 200-mesh copper specimen grid and kept (for 10 min) while the surplus liquid was wiped away using filter paper. The grids were stained with one drop of 3% phosphotungstic acid and then dried for three minutes. The coated grids were observed under a TEM microscope after drying.

### 4.5. Gas Chromatography–Mass Spectrometry (GC-MS)

The effect of the micro fluidization technique applied during the study was investigated by GC-MS analysis. Following Charve et al. [62], a vortex mixer combined 2 mL of the nanoemulsion in a screw-cap vial mixed with 4 mL of diethyl ether. After settling and drying using sodium sulfate anhydrous, a 2 mL screw-cap vial was transferred to the supernatant and wrapped with aluminum foil at 20 °C until analysis. All the extraction steps were repeated in triplicate. A study of the supernatant and hydrodistilled LGEO was conducted by using gas chromatography (Agilent 8890 GC System), coupled to a mass spectrometer (Agilent 5977B GC/MSD) and equipped with an HP-5MS fused silica capillary column (30 m, 0.25 mm i.d., 0.25 mm film thickness). The oven temperature was maintained initially at 50 °C, then programmed from 50 to 200 °C at a rate of 5 °C/min and from 200 °C to 280 °C at a rate of 10 °C/min, then held for 7 min at 280 °C.

Helium was the carrier gas at a 1.0 mL/min flow rate. The injected volume was 1 µL with a split ratio of 1:50. The injection temperature was 230 °C. Electron impact mode (EI) mass spectra were obtained at 70 eV and scanned *m*/*z* range from 39 to 500 amu. The isolated peaks were identified by matching them with data from the mass spectra library (National Institute of Standards and Technology, NIST), standards, and published data. The GC peak areas were used to compute the percentages of the detected constituents. The retention times of a homologous series of C6–C26 n-alkanes were used to determine the Kovats index for each compound, and the values were compared to those found in the literature [63].

### 4.6. Evaluation of LGEO and Its Nanoemulsion Cytotoxicity

#### 4.6.1. MTT Cell Viability Assay

HepG2, WI-38, and Vero cell lines were cultured at a density of 1 × 10^5^ cells/well (100 µL) in Dulbecco’s Modified Eagle Medium (DMEM) with antibiotics (10,000 U of penicillin/10 mg of streptomycin in 0.9% saline) and 10% phosphate-buffered saline serum (PBS). They were kept at 37 °C and 5% CO_2_ for 24 h. A serially diluted oil and nanoemulsion were used to treat the cell lines after 24 h, with concentrations ranging from 0.097 to 1000 µg/mL for HepG2 and 31.25 to 1000 µg/mL for Vero and WI-38 cell lines. A positive control (cisplatin) was used to compare concentrations ranging from 0.01 to 1000 µg/mL. Then, 10 µL of a 12 mM MTT stock solution (5 mg/mL MTT in sterile PBS) was applied to each well. The MTT solution was removed after 4 h of incubation at 37 °C, and the precipitated purple formazan crystal was dissolved in dimethyl sulphoxide (DMSO) for 20 min. A total of 100 µL of an uncultured medium was combined with 10 µL of the MTT stock solution as a negative control. With a BMG LABTECH^®^-FLUOstar Omega microplate reader (Ortenberg, Germany), the absorbance was determined at 540 nm. The proportion of surviving cells was calculated as follows:

[(OD sample − OD blank)/(OD control − OD blank) × 100%](1)

OD sample is the optical density of the sample, OD blank is the optical density of the blank (DMSO), and OD control is the optical density of the control. The curve was illustrated based on the variation of the proportions of surviving cells according to the concentrations, and IC50 was calculated using the obtained sigmoidal curve [64].

#### 4.6.2. WST-1 Cell Viability Assay

Cell viability was assessed by WST-1 assay using an Abcam^®^ kit (ab155902 WST-1 Cell Proliferation Reagent). Aliquots of 50 μL cell suspension (3 × 10^3^ cells) were seeded in 96-well plates and incubated in complete media for 24 h. Cells were treated with another aliquot of 50 μL media containing oil or nanoemulsion at serial concentrations: 3.9 to 1000 µg/mL for HepG2, 31.25 to 1000 µg/mL for Vero and WI-38, and 0.01 to 1000 for cisplatin (positive control). After 48 h of drug exposure, the cells were treated with 10 μL WST-1 reagent, and the absorbance was measured (after 1 h/450 nm) using a BMG LABTECH^®^- FLUOstar Omega microplate reader [65].

#### 4.6.3. Cell Morphology

A 40X Zeiss Axio Vert A1 microscope (Carl Zeiss Microscopy Gmbh, 07745 Jena, Germany) was used to evaluate the morphological alterations in several cell lines subjected to varying concentrations of LGEO and nanoemulsions compared to the control.

### 4.7. Antibacterial Activity of LGEO and Its Nanoemulsion Using Agar Diffusion Method

The antibacterial activity of LGEO and its nanoemulsion was evaluated in vitro on the Gram-positive and Gram-negative bacterial strains described above using the disk diffusion method [17]. The assay was performed in triplicate, and ciprofloxacin was applied as a standard antibiotic.

### 4.8. Evaluation of the Antifungal Effect of LGEO and Its Nanoemulsion

#### 4.8.1. Spore Suspension Preparation for Antifungal Evaluation

Cultures of toxigenic fungal species (7 days old) grown on potato dextrose agar slants at 28 °C were used to prepare the spore suspension. The apparent inoculum suspension with conidia was transferred to a fresh tube, and its optical density was equal to 0.5 McFarland standards. The final inoculum was set from 1.2 to 1.3 × 10^3^ colony forming units per mL (CFU/mL). The preparation of the conidial suspension of fungi conidia was harvested from 7-day-old cultures by pouring a sterile 0.01% aqueous solution of Tween 80 onto the culture plates and scraping the plate surface with a bent glass rod to facilitate the release of conidia. The number of conidia was adjusted to approximately 1.3 × 10^3^ conidia/mL using a Burker–Turk counting chamber [66].

#### 4.8.2. Well Diffusion Test

Applied strains of fungi were reactivated from lyophilized stocks on Czapek-Dox agar media. The activated strains were spread on Czapek-Dox agar plates, and the disks or wells were loaded using 100 μL of oil and its nanoemulsion. The impact of the applied extract on the inhibition was recorded as a clear zone diameter (mm) for each strain: the more effective the inhibition zone diameter, the more influential the concentration. A standard antifungal material (Nystatin) was utilized as a positive control material [64].

#### 4.8.3. Determination of Minimal Antifungal Concentrations (MFC)

The MFC was evaluated according to the method described by Badr et al. [67]. According to the Clinical Laboratory Standards Institute (approved standard M38-A2 guidelines), the broth microdilution technique was utilized to investigate antifungal susceptibility. Nystatin, a standard fungicidal, was applied as a positive control in this study.

#### 4.8.4. Simulated Experiment to Evaluate the Anti-Mycotoxigenic Impact

Two of the above-evaluated strains were selected for the following study evaluations: *A. parasiticus* ITEM 11 and *A. carbonarius* ITEM 5010. The strains reactivated on the yeast extract sucrose (YES) media to the acclamation step. After two reactive steps, a known concentration of *A. parasiticus* and *A. carbonarius* (1.3 × 10^3^) was utilized for the experiment. However, *A. parasiticus* and *A. carbonarius* are known to produce aflatoxins and ochratoxin A, respectively.

The investigation steps were carried out to evaluate the LGEO and MF-LGEO impact against the fungal spores using the simulated media. Briefly, 5 mL of a spore suspension of each fungus was added to 250 mL media of the YES in conical flasks individually. At the end of the incubation time (10 days/28 °C), media were filtered using Whatman No.1 (known weight for each filter). The collected filters were then dried using a hot air oven (45 °C/1 d) until a constant weight was achieved for each [64]. The mycelial mass was recorded, and the treatment growth weight was calculated as a ratio of the control growth weight using the following equation:

(2) $$\%\;Mycelial\;growth\;weight=\left(1-\frac{M1-M2}{M1}\right)\times 100$$

where *M*1 is the weight of the control dry mycelia, and *M*2 is the weight of the treated dry mycelia.

### 4.9. Determination of Mycotoxin Degradation in Simulated Media

Utilizing the filtrated media resulting from the previous step, the AF content was extracted and estimated in the growth media of the *A. parasiticus*. The AOAC-approved technique was used for the extraction step [68]. The culture broth (10 mL) was mixed twice with 10 mL chloroform, shaking vigorously for about 10 min, and then separated using a separating funnel. The lower phase was dried over anhydrous extra-pure sodium sulfate, evaporating the chloroform in nitrogen. HPLC-grade acetonitrile was used to dissolve the dry film. One milliliter of the solution was combined with 10 mL of distilled water before being placed in an Afla-test^®^ immune affinity column and washed twice with 10 mL of distilled water (flow rate: 6 mL/min). A total of 2 mL of acetonitrile (flow rate: 0.3 mL/min) was used to elute the AFs. The quantitative analyses were performed using a pre-calibrated fluorometer (VICAM Series™, 4EX Fluorometer, Watertown, MA, USA; the LOD 1.0 μg/L).

The extracted media of *A. carbonarius* was utilized for ochratoxin A determination of the control, LGEO, and MF-LGEO impacts. The OCA was extracted from the culturing broth using immunoaffinity columns (OchrA-test^®^ from Biomin). Fifty milliliters of each culture broth were combined with 200 mL aqueous acetonitrile (60%) (*v*/*v*) and swirled for 2 min at high speed before filtering through a Whatman No. 3 filter paper and a microfiber filter. To reach a solvent concentration of 2.5%, about 4 mL of the resultant extract was diluted with 44 mL of phosphate-buffered saline (PBS, pH 7.4). The mixture was gravity-passed through the OchrA-test column (5 mL/min). The column was washed with 20 mL of PBS before eluting OCA with 1.5 mL of acetic acid: methanol combination (2:98, *v*/*v*) and 1.5 mL of clean water. The quantitative analyses were performed using a pre-calibrated fluorometer (VICAM Series™, 4EX Fluorometer, Watertown, MA, USA; the LOD 1.0 μg/L).

### 4.10. Statistical Analysis

Using Graph Pad Prism 7 (Graph Pad Software Inc., San Diego, CA, USA), statistical data analysis was carried out. The findings were presented as means ±standard deviations (SD) based on at least three replicates. The significance of the difference between the mean values was determined using analysis of variance (ANOVA), and Duncan’s multiple range test was computed (*p* = 0.05).

## 5. Conclusions

The LGEO terpenes showed enhanced content by the microfluidization process, including γ-Terpinene, neral, geranial, and carvacrol, as well as a new record for decanal content. Characteristics of a prepared microfluidization emulsion of MF-LGEO showed a distinctive droplet diameter at 20.76 ± 0.36 nm and a PDI of 0.179 ± 0.03. The nanoemulsion value of ξ-potential emphasized the emulsion stability. Cytotoxicity of LGEO and MF-LGEO against three cell-line strains recorded promising values determined by two assays (MTT and WST-1). Also, the ameliorative effect of the microfluidization process is recorded regarding the cell line experiment with an anti-inflammatory effect increment. These results are illustrated and linked to the component changes recorded by the GC-MS analysis for the MF-LGEO. By evaluating the antimicrobial effect, the result reflects a greater potency of MF-LGEO than LGEO against pathogenic strains of Gram-positive and Gram-negative bacteria. The activity amelioration joined to the change in essential content using the microfluidization technique. The LGEO and its microfluidization oil were noticed to have antifungal activity against mycotoxigenic strains of fungi. Again, the microfluidization process enhanced the anti-mycotic and anti-mycotoxigenic impact of LGEO against the toxigenic strains of fungi. These results recommend the promising MF-LGEO as a solution against toxigenic fungal contamination, which can reduce the toxin production of these fungi. More investigation is needed to fully understand the anti-mycotic and anti-mycotoxigenic effects of MF-LGEO by its food application.

## Figures and Tables

**Table 1:** Volatile constituents’ identification of the LGEO and MF-LGEO using GC-MS.

S/N	Compound	RI ^a^	LRI ^b^	Area%		Identification Method ^c^
				LGEO	MF-LGEO	
**1**	6-Methyl-5-heptene-2-one	983	985	0.94	0.59	RI, MS
**2**	*β*-Myrcene	992	991	3.61	2.76	RI, MS, STD
**3**	*Z-β*-Ocimene	1040	1037	0.64	0.52	RI, MS
**4**	*E-β*-Ocimene	1051	1050	0.34	0.70	RI, MS
**5**	*γ*-Terpinene	1063	1059	0.48	0.81	RI, MS
**6**	Linalool	1100	1096	1.42	0.98	RI, MS, STD
**7**	Perillene	1105	1103	0.31	-	RI, MS
**8**	*trans*-Pinocarveol	1140	1139	0.42	0.42	RI, MS
**9**	Camphor	1145	1146	0.47	-	RI, MS
**10**	Citronellal	1156	1153	0.34	-	RI, MS
**11**	Isoneral	1171	1170	1.35	0.85	RI, MS
**12**	Rose furan oxide	1180	1177	0.65	0.36	RI, MS
**13**	Isogeranial	1189	1185	2.07	1.44	RI, MS
**14**	Decanal	1204	1201	-	1.57	RI, MS
**15**	Citronellol	1228	1225	2.10	1.42	RI, MS, STD
**16**	Neral	1240	1238	26.91	28.95	RI, MS, STD
**17**	Geraniol	1258	1255	9.69	7.97	RI, MS, STD
**18**	Geranial	1270	1267	30.73	35.48	RI, MS, STD
**19**	Dihydrolinalool acetate	1279	1275	1.41	1.88	RI, MS
**20**	Carvacrol	1298	1299	2.61	3.95	RI, MS, STD
**21**	Nerolic acid	1337	1340	0.97	-	RI, MS
**22**	Geranic acid	1351	1355	1.14	0.51	RI, MS
**23**	Neryl acetate	1365	1361	2.19	1.36	RI, MS
**24**	Geranyl acetate	1384	1383	5.06	4.55	RI, MS
**25**	Bergamotene (*α*-trans-)	1438	1434	0.33	-	RI, MS
**26**	*α*-Farnesene	1508	1505	0.43	-	RI, MS
**27**	Caryophyllene oxide	1582	1583	0.47	-	RI, MS
**28**	Selin-6-en-4α-ol	1633	1636	0.65	0.46	RI, MS
	Total	-	-	97.73	97.53	-

**RI ^a^**: Retention indices calculated on DB-5 column using alkanes standards. **LRI ^b^**: Retention indices according to the literature. **^c^** Confirmed by comparison with the retention indices, the mass spectrum of the authentic compounds, and the NIST mass spectra library data. LGEO: lemongrass emulsion of essential oil; MF-LGEO: microfluidizing emulsion of lemongrass essential oil.

**Table 2:** Cytotoxic activity of LGEO and its microfluidized nanoemulsion against HepG2, Vero, and WI-38 cell lines using MTT and WST-1 assays.

Cell Line	LGEO (IC_50_ μg/mL)		MF-LGEO (IC_50_ μg/mL)		Cisplatin (Control) (IC_50_ μg/mL)	
	MTT	WST-1	MTT	WST-1	MTT	WST-1
HepG2	1.78 ± 0.08	28.54 ± 2.26	230.77 ± 3.12	249.08 ± 2.77	20.71 ± 1.15	40.95 ± 1.88
WI-38	-	-	618.65 ± 5.61	957.41 ± 7.11	277.6 ± 4.5	401.2 ± 3.66
Vero	236.91 ± 5.2	111.04 ± 6.76	-	-	142.33 ± 4.12	287.6 ± 3.43

The data are expressed as means ± SD (where n = 3, *p* ≤ 0.05); SD: standard deviation. LGEO: lemongrass emulsion of essential oil; MF-LGEO: microfluidizing emulsion of lemongrass essential oil.

**Table 3:** Lemongrass oil and its nanoemulsion impacts on toxigenic fungal growth and mycotoxin production of *A. flavus* and *A. carbonarius* germinated spores in simulated media.

Treatment	Mycelia Weight	Growth (%)	MFC (mg/mL)	Total AFs (ng/mL)	Reduction (%)	OCA (ng/mL)	Reduction (%)
*A. flavus* fungi							
Control	6.1848 ± 0.424	100	-	922.71 ± 20.32	-	-	-
LGEO	3.6949 ± 0.588	59.74	1.76	509.11 ± 20.02	44.82	-	-
MF-LGEO	1.6418 ± 0.418	26.54	0.81	421.67 ± 18.66	54.3	-	-
Nystatin	0.9945 ± 0.117	16.07	0.05	106.81 ± 12.41	88.42	-	-
(100 µg/mL)							
*A. carbonarius* fungi							
Control	5.3477 ± 0.477	100	-	-	-	414.33 ± 7.32	-
LGEO	2.9971 ± 0.686	56.04	1.45	-	-	221.52 ± 8.29	46.53
MF-LGEO	1.2616 ± 0.284	23.59	0.65	-	-	105.37 ± 5.54	74.57
Nystatin	1.0057 ± 0.225	18.81	0.03	-	-	67.81 ± 5.08	83.63
(100 µg/mL)							

The data are expressed as means ± SD (where n = 3, *p* ≤ 0.05); SD: standard deviation; MFC: minimal fungicidal concentration. LGEO: lemongrass essential oil; MF-LGEO: microfluidizing nanoemulsion of lemongrass essential oil. Growth (%) represents the fungal growth ratio compared to the fungal growth of the control (without treatment in media). Reduction (%) represents the decreases of toxin production in the media containing the treatment compared to toxin content in the control media (without treatment in media).

## Data Availability

All the data regarding the present manuscript are included. All data linked to the present manuscript are included; no other related data are available.

## Supplementary Materials

The following supporting information can be downloaded at: https://www.mdpi.com/article/10.3390/molecules28145367/s1. Figure S1. TEM images of microfluidized LGEO nanoemulsion; Figure S2. cytotoxicity impact of LGEO and MF-LGEO on HepG2, WI38, and Vero cell lines at different concentrations

## Sample Availability

Not available.
