# Supplementary material for: Microfluidizing Technique Application for Algerian Cymbopogon citratus (DC.) Stapf Effects Enhanced Volatile Content, Antimicrobial, and Anti-Mycotoxigenic Properties
Source: Molecules. 2023 Jul 12;28(14):5367. doi: 10.3390/molecules28145367 (PMC10384219; doi:10.3390/molecules28145367)
Supplement: Supplementary file 1 [file molecules-28-05367-s001.zip › molecules-2480515-supplementary.pdf]

(a) **(b)**

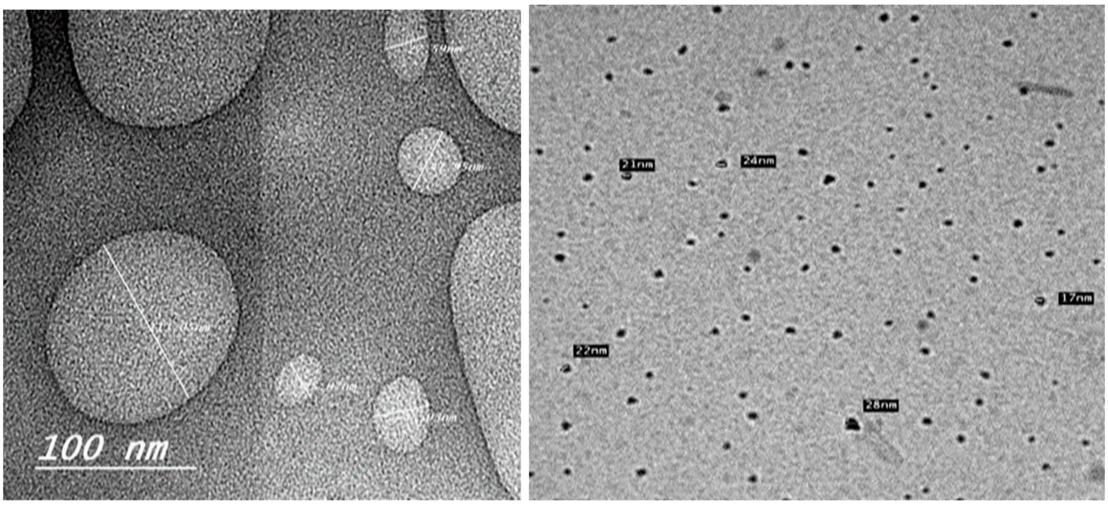

Figure S1. TEM images of microfluidized LGEO nanoemulsion

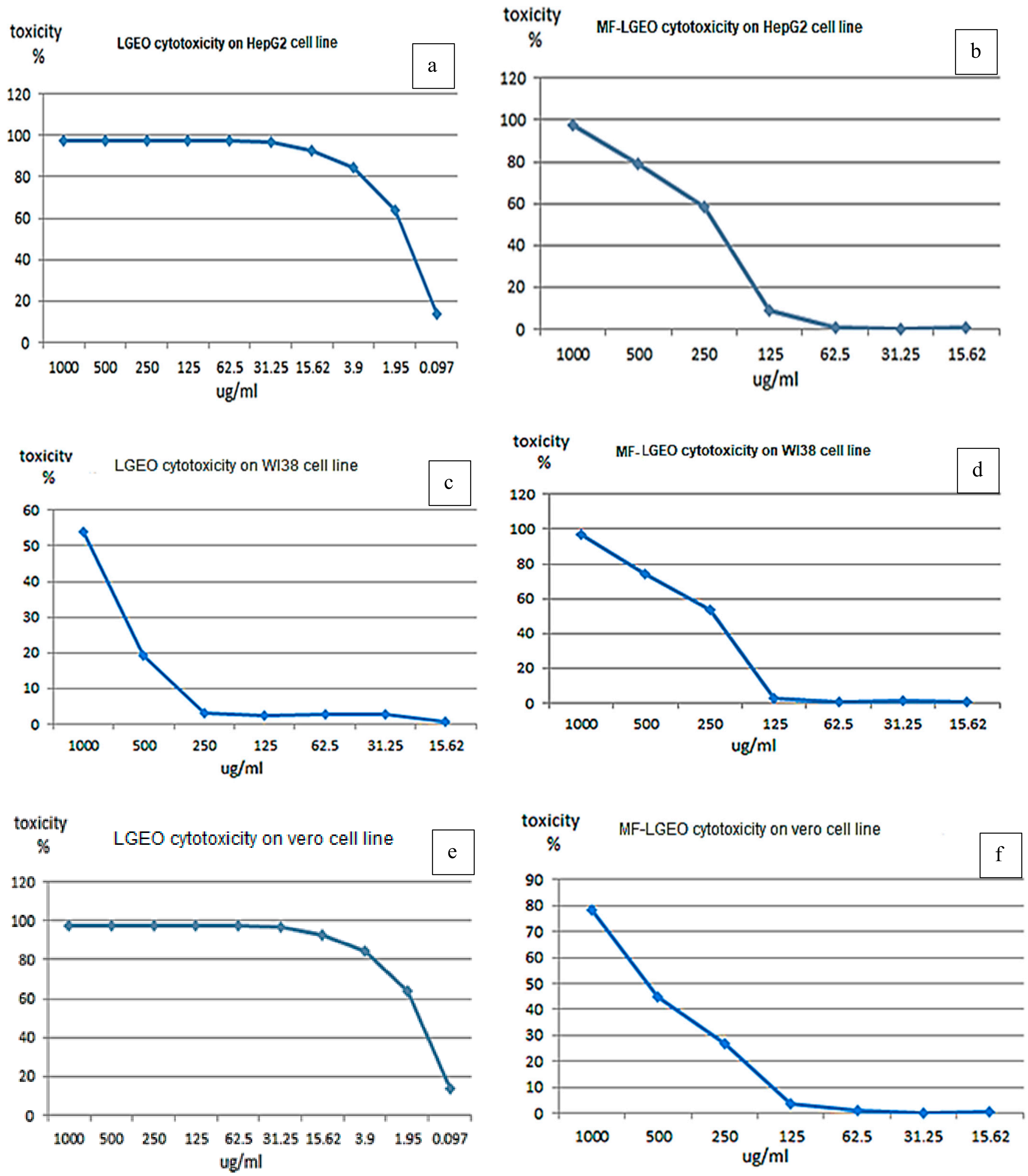

Figure S2. Cytotoxicity impact of LGEO and MF-LGEO on HepG2, WI38, and Vero cell lines at different concentrations.
